# Size and space controlled hexagonal arrays of superparamagnetic iron oxide nanodots: magnetic studies and application

**DOI:** 10.1038/srep02772

**Published:** 2013-09-27

**Authors:** Tandra Ghoshal, Tuhin Maity, Ramsankar Senthamaraikannan, Matthew T. Shaw, Patrick Carolan, Justin D. Holmes, Saibal Roy, Michael A. Morris

**Affiliations:** 1 Materials Research Group, Department of Chemistry and Tyndall National Institute, University College Cork, Cork, Ireland; 2 Centre for Research on Adaptive Nanostructures and Nanodevices (CRANN), Trinity College Dublin, Dublin, Ireland; 3 Micropower-Nanomagnetics group, Tyndall National Institute, Cork, Ireland; 4 Intel Ireland Ltd., Collinstown Industrial Estate, Co. Kildare, Ireland; 5 Current address: Electrical Engineering Department, Stanford University, California, USA (on sabbatical from Tyndall National Institute, Ireland)

## Abstract

Highly dense hexagonally arranged iron oxide nanodots array were fabricated using PS-b-PEO self-assembled patterns. The copolymer molecular weight, composition and choice of annealing solvent/s allows dimensional and structural control of the nanopatterns at large scale. A mechanism is proposed to create scaffolds through degradation and/or modification of cylindrical domains. A methodology based on selective metal ion inclusion and subsequent processing was used to create iron oxide nanodots array. The nanodots have uniform size and shape and their placement mimics the original self-assembled nanopatterns. For the first time these precisely defined and size selective systems of ordered nanodots allow careful investigation of magnetic properties in dimensions from 50 nm to 10 nm, which delineate the nanodots are superparamagnetic, well-isolated and size monodispersed. This diameter/spacing controlled iron oxide nanodots systems were demonstrated as a resistant mask over silicon to fabricate densely packed, identical ordered, high aspect ratio silicon nanopillars and nanowire features.

The number of magnetic material types is limited and, thus, the range of magnetic properties (coercivity, saturation magnetization, remanance, etc.) is also limited^1^,^2^. The demands for more advanced magnetic materials has largely been driven by the information and communications technology (ICT) industries for uses in e.g. data storage/memory, power applications and even as potential replacement of CMOS logic devices^3^,^4^,^5^. Two trends have emerged. First, to develop materials on-chip as part of complex silicon circuitry and secondly, nanostructuring of the magnetic materials (i.e. patterning) to reduce dimension and allow device miniaturization and densification. However, nanopatterning of magnetic materials and, in particular, films is a challenge as they are not readily or simply etched and films grown on silicon tend to suffer damage and delamination during conventional processing^6^. The magnetics used tend to be complex mixtures and can suffer composition changes during processing^7^. In these ways, integration of magnetics with conventional silicon devices remains a significant challenge.

As an alternative to conventional photolithographic processing, self-assembly has been often explored as a potential technique to create patterned magnetic substrates^8^,^9^. However, these have been limited in application because attaining the required size and shape uniformity providing required mechanical robustness of samples has proved difficult^10^,^11^. We have recently shown that the microphase separation of block copolymers can provide a platform to simple and complex oxide materials at surfaces by a process of selected block inclusion^12^,^13^. Iron oxide provides a suitable exemplary to work on since it is provides a system of choice in many applications^14^,^15^,^16^. In this work, we wanted to develop this approach to demonstrate that it could be used to control the size of the oxide particles and their distance apart as well as the technique being compatible with further silicon based processing to yield complex heterostructures. To study the magnetic this effect we have shown the formation of structurally and compositionally well-defined iron oxide nanodot arrangements. And used conventional silicon based etch processing to create magnetic oxide phases supported on silicon pillar structures.

The work also provided us with a platform of the magnetic properties of these nanodot arrangements. The understanding of these systems needs to be extended because of their potential applications in ICT and biological areas^1^,^3^. However, quantitative study of these systems is difficult because the generally used chemical synthesis methods are challenged in producing ultra-small particles that are size/shape monodispersed and are uniformly dispersed in solution or on a substrate^17^,^18^. Further, defining the relationship between nanoparticle crystal structure and their magnetic properties is difficult because of the dimensional non-uniformity and using diffraction techniques for the study of very small dimensions. As above, nanofabrication of ultra-small dimension magnetic nanostructures at substrates by lithography is also challenging^19^,^20^. Thus, we have used these ‘engineered’ nanooxide patterns to explore the magnetic properties of nanodimensioned particles and also the effect of inter-particle interactions. This has allowed us to properly quantify important parameters that define the magnetism in these systems.

In this work we combine emerging methods in block copolymer lithography with solution based nanoparticle preparation to fabricate nanodots arrays of controlled size and separation. In this way it is shown that the particles not only demonstrate superparamagnetism but also the variation in magnetic properties with size and spacing in the nanoscale can be described precisely. Recently, iron oxide nanodots was proven as an excellent resistant mask over silicon through the ICP etch process^21^. By combining a dry etch technique and using the oxide nanodots as a hard mask, the high density nanopillars/wires can be generated with controlled distance and topography ensuring complete separation.

## Results

### Dimensional and structural control of block copolymer nanopatterns

**Figure 1:**
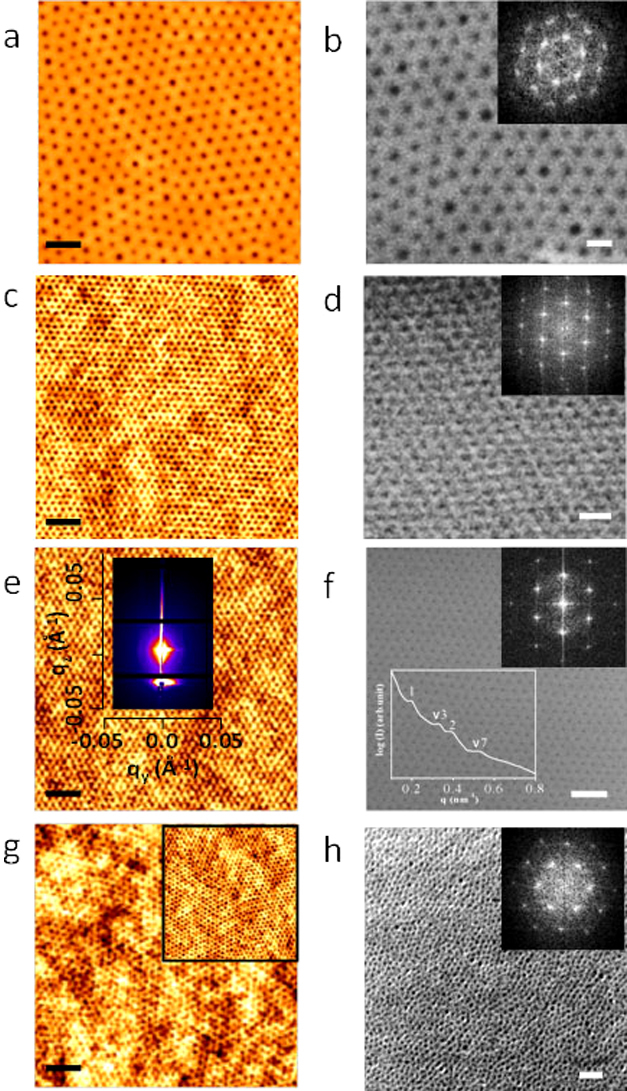
AFM and SEM images of microphase separated dot patterns after solvent annealing for different molecular weight PS-PEO systems (a), (b) 102 k–34 k, (c), (d) 42 k–11.5 k, (e), (f) 32 k–11 k and (g), (h) 16 k–5 k respectively. Insets of (b), (d), (f), (h) represents corresponding FFT patterns. Insets of (e), (f) shows GISAXS 2D pattern and intensity profile with respect to the peak position. (a), (c), (e), (g) scale bar: 200 nm. (b), (d), (f), (h) scale bar: 100 nm.

The dimensional control over the self-assembled block copolymer nanopatterns was achieved by different molecular weight PS-PEO (polystyrene-b-polyethylene oxide) systems and the corresponding compositions of the constituent blocks represented as S1 (102 k–34 k), S2 (42 k–11.5 k), S3 (32 k–11 k) and S4 (16 k–5 k). The as-coated films of S1, S2 and S3 exhibited little indication of periodic ordering. Poorly ordered microphase separation without any controlled orientation was observed for S4. A solvent annealing process was used to achieve vertically oriented cylindrical PEO microdomains in the PS matrix. The coated films were annealed at a temperature 50°C either in toluene or in toluene/water mixed solvents to control the structural arrangement of the cylindrical domains depending on the block compositions. Figure 1 shows the representative tapping mode AFM and SEM images of the PS-b-PEO systems after the solvent exposure and indicates ordered arrangements over large areas with no indication of de-wetting. In the AFM images, the PEO cylinders were darker in colour. The SEM can resolve the PS and PEO microdomains despite their similar densities and average atomic number^22^. The films are of regular thicknesses of 45 nm, 40 nm, 25 nm and 35 nm for S1, S2, S3 and S4 respectively. The corresponding average centre to centre distances between adjacent microdomains are 90 nm, 42 nm, 32 nm and 25 nm whereas the PEO cylinder diameters were 38 nm, 19.3 nm, 17 nm and 11 nm respectively (Figures 1a–h). The intense spots in the FFT pattern shown in the insets of the SEM images (Figures 1b, d, f, h) confirms the hexagonal arrangement of PEO cylinders. Similar patterns were found on different substrates such as glass, quartz, silica etc. The large scale ordering, uniformity and feature size suggest applicability in the area of addressable media where the spatial positioning of each element must be defined to within a fraction of repeat period.

Indicative GISAXS 2D scattering profile of the S3 after solvent annealing is shown in the inset of Figure 1e. The critical angle of silicon was determined to be 0.2° from X-ray reflectivity data. At an incident angle 0.2°, the incident beam penetrates through the entire film and the image exhibits multiple reflections along the q_y_ scattering vector indicating the ordered periodic structure of the film. From the detailed q_y_ line scan profile, the first order peak is observed at q_y_ = 0.0195 
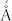
^−1^, which corresponds to a microdomain spacing 32.2 nm consistent with the AFM and SEM results (inset of Figure 1f). The hexagonal packing of the cylindrical PEO microdomains is confirmed by the relative peak positions of higher order reflections with respect to the first peak position, yielding the expected values 1, √3, 2 and √7.

### Creation of nanoporous templates

**Figure 2:**
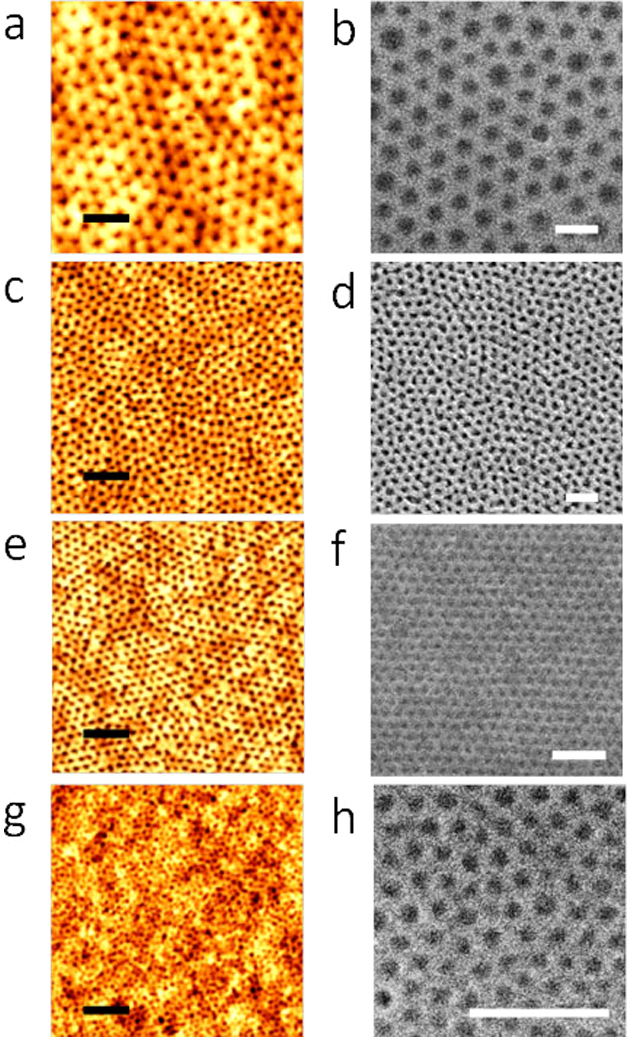
AFM and SEM images of the nanoporous template after ethanol ultrasonication for different periods of time (a), (b) 102 k–34 k, 20 min (c), (d) 42 k–11.5 k, 17 min (e), (f) 32 k–11 k, 15 min and (g), (h) 16 k–5 k, 10 min respectively. (a), (c), (e), (g) scale bar: 200 nm. (b), (d), (f), (h) scale bar: 100 nm.

In order to generate identical order inorganic oxide nanostructures from the microphase separated PS-b-PEO thin film, it was necessary to effectively etch the minor component, PEO, to generate the long range ordered template for inorganic inclusion. This was achieved by chemical degradation and/or modification of the PEO block through ultrasonication of the film in ethanol. The structural periodicity and dimensions are essentially unchanged after ethanol treatment as revealed by AFM and SEM images in Figure 2. The optimized ultrasonication times are 20 min, 17 min, 15 min and 10 min for S1, S2, S3 and S4 respectively. Presumably, the reduction in the optimum time as molecular weight decreases is related to reduce mass transport limitations at smaller dimensions. Note that the ethanol exposure had to be carefully optimized as longer exposures/higher temperatures resulted in surface roughness or structural degradation of the film. Following ethanol exposure, all the images showed an increment in the phase contrast without affecting the long range order. Further, the cylinder to cylinder spacings and the PEO cylinder diameters remained unchanged. No thickness change was observed as measured by ellipsometry. No deformation or discontinuity of the nanoporous template was observed^23^. Thus, it is possible to apply this simple and effective PEO degradation process to a wide range of PS-PEO systems.

### Structural arrangement of the nanoporous templates

**Figure 3:**
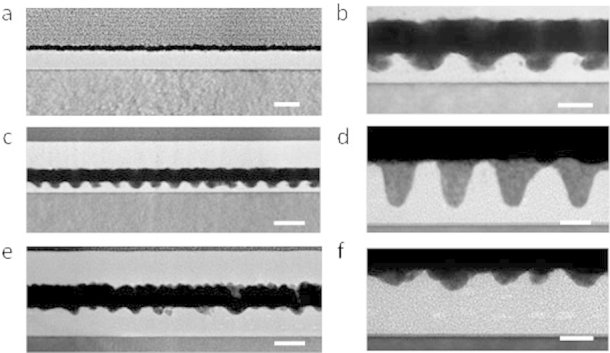
Cross-sectional TEM images of (a) solvent annealed film for 16 k–5 k and nanoporous template after ethanol ultrasonication for different time (b), (c) 32 k–11 k, 15 min (d) 16 k–5 k, 10 min (e), (f) 16 k–5 k, 5 min respectively. (a), (c), (e) scale bar: 50 nm. (b), (d), (f) scale bar: 20 nm.

The effect of the ethanol treatment is difficult to assess by top-down SEM imaging but is of importance if the templating role of the films is to be understood. Here we used the deposition of gold during TEM lamellae preparation to provide contrast enhancement and provide mechanical rigidity. Prior to ethanol treatment, lack of contrast produces a featureless image of the film (Figure 3a). However, Figures 3b–3d reveals the PEO cylinder arrangements within the film after selective removal of the PEO block. The polymer template is well adhered to the surface with no indication of deformation or delamination. The ethanol ultrasonic treatments results a smooth film surface and the data suggest that it largely affects the PEO component. For each film, the ‘nanopores’ formed are of a consistent depth across the film. Higher magnification images of S3 in Figure 3b gave the diameters and depths of the nanopores at 17 and 18 nm respectively. These pores do not penetrate to the surface and a 7 nm non-porous PS layer exists on top of the Si substrate as has been suggested before^12^. Similar data were obtained for the sample S4 after the optimized 10 min ethanol ultrasonication (Figure 3d). The TEM derived thicknesses are consistent with the ellipsometry measurement supporting the suggestion that PS is not affected. For S4, the diameters and depths of the PEO derived regions were 11 nm and 26 nm respectively and the PS wetting layer at the substrate was 9 nm thick.

To illustrate the need to optimize the ethanol treatment periods, Figures 3e and 3f show TEM cross-sections for sample S4 reducing the ultrasonication time to 5 min. The image suggests only partial removal of the PEO microdomains starting from the top surface of the film. Layers with a hexagonal arrangement of PEO cylinders are also just visible (elliptical regions of paler contrast) along the thickness of the film. It should be noted that the ethanol PEO removal process is not linearly dependent with time as can be seen in comparing the pore depth between Figures 3d and 3f. It is suggested that this is because the PEO removal process includes two steps; swelling of the PEO block followed by solvation with the former being kinetically limited.

### Surface composition by FTIR and XPS

**Figure 4:**
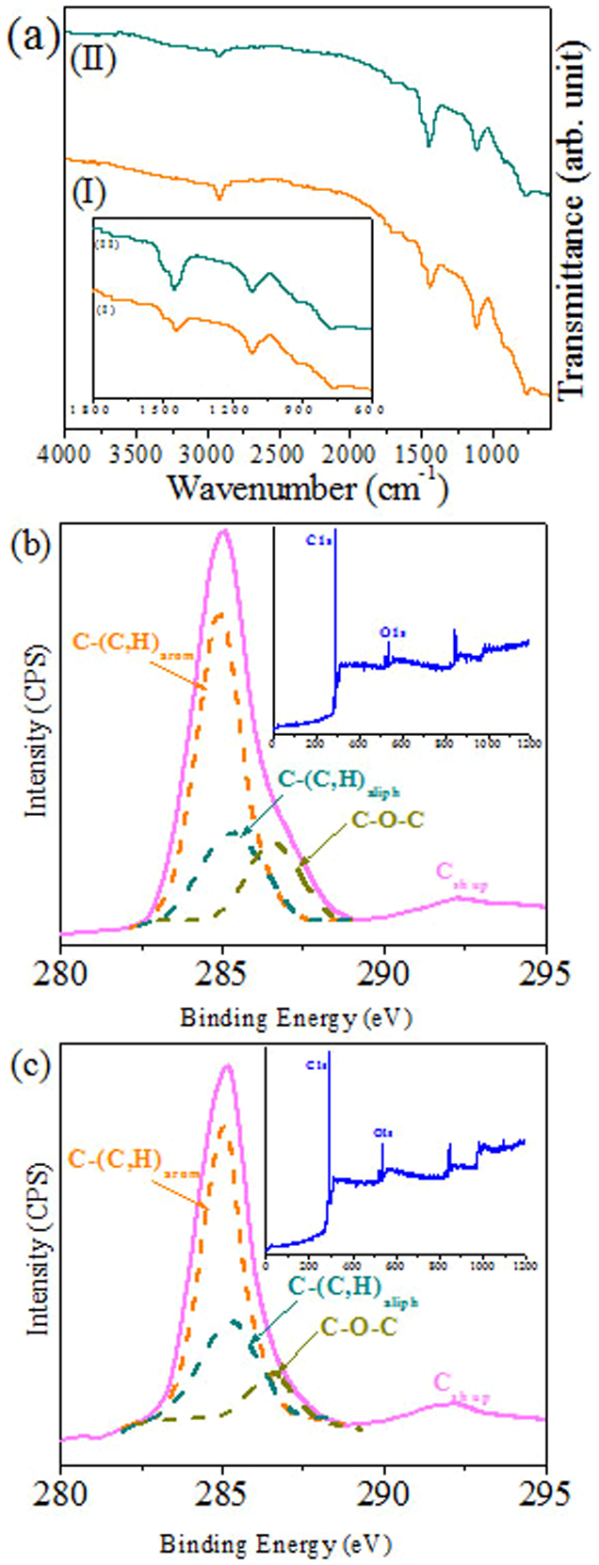
(a) FTIR and (b), (c) C1s core level spectra of the PS-PEO hexagonal dot patterns before and after ethanol treatment for 32 k–11 k. Insets of (b and c) show corresponding survey spectra.

FTIR data also provide additional information on the ethanol ultrasonic treatment. FTIR transmittance spectra of the solvent annealed and ethanol treated film for sample S3 is shown in Figure 4a. Both films show features typical of the PS and PEO blocks. Peaks at 768 cm^−1^ (benzene bending) and 1608 cm^−1^, 1496 cm^−1^ and 1454 cm^−1^ (benzene ring stretching), weak overtone and combination bands in the range of 1655–2000 cm^−1^ all be attributed to polystyrene^24^. The features at 1108 cm^−1^ (C-O-C stretch), 929 cm^−1^ (CH_2_ PEO rocking modes), and 1752 cm^−1^ and 1719 cm^−1^ (C = O stretches of the ester and keto group respectively) as well as peaks at 2927 cm^−1^ and 2854 cm^−1^ (CH_2_ PEO stretching modes) can all be assigned to the PEO block^25^. A broad band in the range of 3300–3700 cm^−1^ also appears due to alcohol type O-H stretching vibrations and/or atmospheric moisture. It is observed that the relative intensity of the PS to the PEO increases for the ethanol treated film (Figure 4a(II)) as well as the reduction in the band around 3500 cm^−1^. The data suggest that not all of the PEO is removed by the ethanol treatment and the pores formed do contain PEO in a very low density structure not revealed by microscopy.

XPS analyses were performed to confirm the surface composition for PS-PEO films before and after ethanol treatment. All samples displayed similar XPS characteristics. The C1s curve-fitted peaks of sample S3 reveal four components as illustrated in Figures 4b and 4c. Two of these can be attributed to carbon from the aromatic ring of PS (**C**-(C,H)_arom_) and the aliphatic backbone of PS (**C**-(C,H)_aliph_) at 284.9 eV and 285.2 eV respectively. A distinct high binding energy shoulder on the primary C1s peak can be seen particularly for the untreated sample and this is assigned to carbon involved in an ether link (**C**-O-**C**) from PEO at about 286.5 eV. A shake-up satellite assigned to the aromatic ring of PS (**C**_sh up_) at about 292 eV was also seen. The contribution of PEO component to the total C1s peak area intensity decreases from 16% to 9% after the ethanol treatment. This is consistent with loss of some of the PEO. The XPS survey spectra shown in the inset of Figures 4b and c demonstrate a slight increase in the oxygen peak intensity for the ethanol treated film but this may be due to trapping of ethanol within the remaining PEO due to strong hydrogen bonding.

### Generation of iron oxide nanodots array: spatial and dimensional control

**Figure 5:**
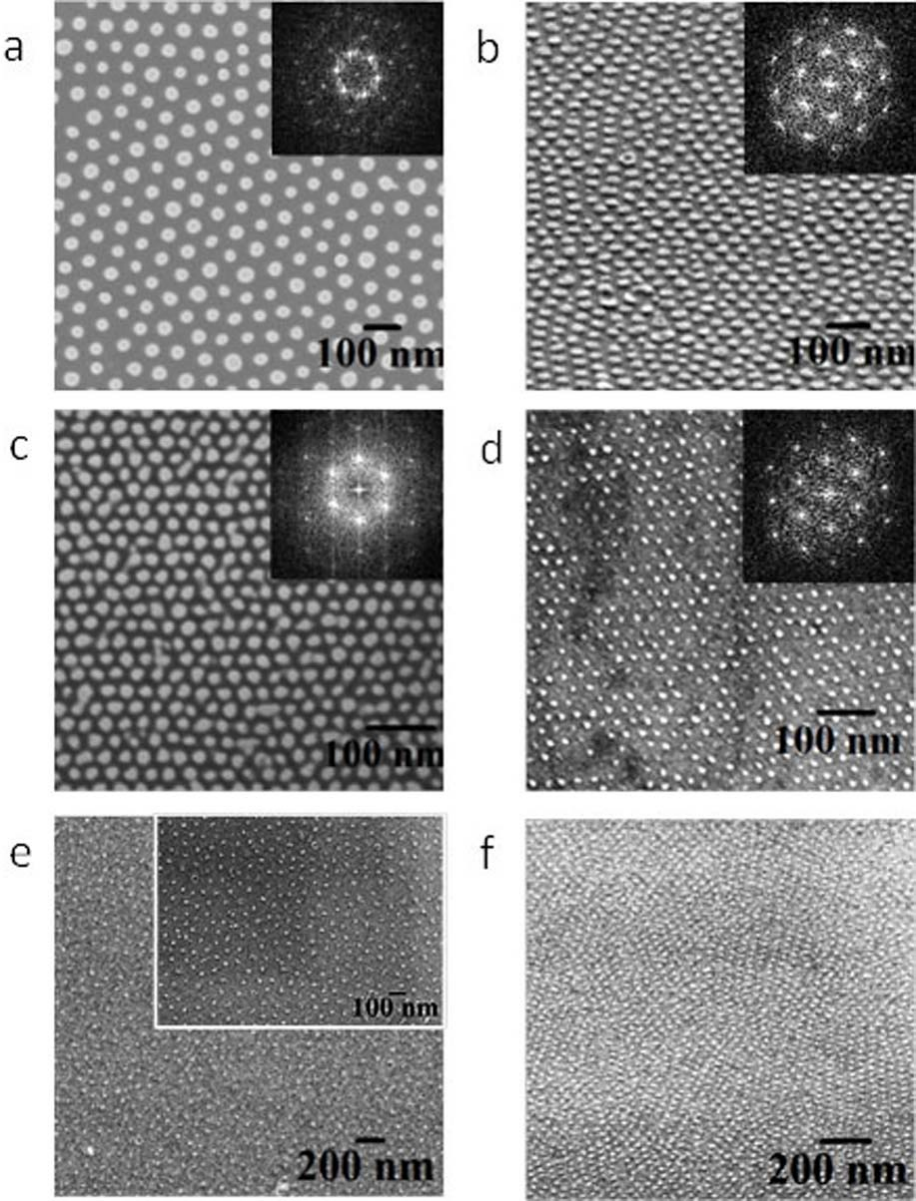
SEM images of the iron oxide nanodots formed after UV/Ozone treatment for different molecular weight systems and with varying concentrations of precursor solution (a) 102 k–34 k, 1 wt%, (b) 42 k–11.5 k, 0.4 wt%, (c) 32 k–11 k, 0.35–wt%, (d) 16 k–5 k, 0.2 wt%, (e) 102 k–34 k, 0.7 wt% and (f) 32 k–11 k, 0.3 wt% respectively. Insets of (a), (b), (c), (d) show the corresponding FFT patterns.

Conventional block copolymer lithography is based around the selective removal of one block and subsequent use of the remaining block as an on-substrate mask. However limitations in etch contrast imposed challenges and limitations^26^. An alternative methodology is to use the DBCP nanopattern to create a hard mask (i.e. a material with very high etch resistance compared to the substrate). In our previous work^21^, it was reported that iron oxide can be an excellent resistant mask for high aspect ratio silicon substrate patterning. This methodology was developed further here. The oxide nanodots were formed by selective metal ion inclusion (via spin-coating of ethanolic solution) coupled to a subsequent UV-Ozone treatment. Figure 5 shows the SEM images of well ordered iron oxide nanodots arrays with various diameters and spacings formed following UV/Ozone treatment. In each sample, the nanodots had uniform size/shape and their placement mimics the original self-assembled block copolymer patterns. The average diameters of the nanodots were 40 ± 4 nm, 24 ± 3 nm, 18 ± 2 nm, 12 ± 2 nm for S1, S2, S3 and S4 respectively when 1 wt%, 0.4 wt%, 0.35 wt% and 0.2 wt% precursor solutions were used (Figures 5a–d). The reduction in solution concentration clearly reflects the pore volume decrease for the films. The average nanodot heights were between 6–10 nm as measured by ellipsometry. The FFT patterns shown in the inset of the figures confirms the hexagonal ordering of the nanodots. The density of the nanodots on the substrate is measured approximately 1.8 × 10^8^, 4.2 × 10^10^, 1.1 × 10^11^ and 6.7 × 10^11^ nanodots cm^−2^. Previous observation suggests that the crystalline nanodots are well adhered to the substrate and thermally robust^13^. The as-prepared phase of iron oxide is Fe_3_O_4_ which transformed upon annealing to Fe_2_O_3_^12^. The crystallinity and phases of iron oxide nanodots before and after annealing is documented in supporting information. An effect of heating was a reduction in the average diameter and height consistent with high temperature densification.

The diameter of the nanoparticles can be further controlled by varying the concentrations of the precursor solution without changing the spacing between them. For example, Figure 5e shows the SEM image of well ordered, 25 ± 2 nm (diameter), 90 nm spaced, iron oxide nanodots derived from S1 using a 0.7 wt% precursor concentration (compared to 40 nm from a 1.0 wt% solution as above). The ordering or uniformity is not compromised at the lower concentration used as depicted in the inset of Figure 5e. Similarly for sample S3, nanodots of 15 ± 2 nm diameter (18 nm for 0.35 wt%) was prepared (Figure 5f). Different concentrations of the iron precursor solution altered the nanodot diameter as well as the thickness of the resultant nanodots (which is clearly an important parameter). As the precursor solution prepared typically consists of a large volume fraction of ethanol, the free volume of the cylinders is predominantly filled by ethanol, thus, the quantity of the inorganic component within the PEO cylinders depends on the concentration of the precursor used. In this way, the diameter and thickness of the nanodots are related to the precursor concentration. Note that a narrow size distribution is observed for smaller to moderate concentrations of precursors for a particular molecular weight of block copolymer compared to higher concentrations of precursors. Thus, highly dense, size controlled uniform hexagonal array of iron oxide nanoparticles can be prepared with maintenance of their inter-distance and long range ordering on the solid substrate.

### Fabrication of Si nanopillars and nanowires

**Figure 6:**
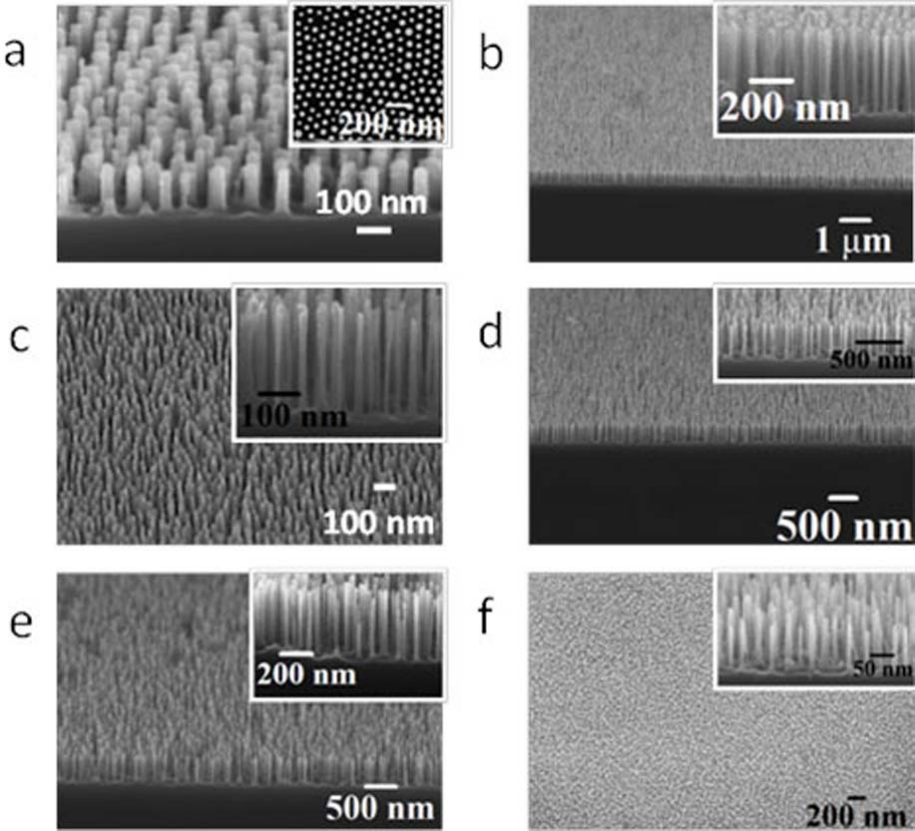
Tilted and cross-sectional SEM images of the Si nanopillars/wires after pattern transfer into the Si substrate for different molecular weight systems and with different etch time (a) 102 k–34 k, 3 min, (b) 42 k–11.5 k, 10 min, (b, inset) 42 k–11.5 k, 8 min, (c) 32 k–11 k, 5 min (d) 32 k–11 k, 8 min, (e) 32 k–11 k, 10 min and (f) 16 k–5 k, 3 min respectively.

These ordered iron oxide nanodots (Fe_2_O_3_) were used to fabricate densely packed features on Si substrate as previously demonstrated by us for the S2 material^21^. Below, we demonstrate the methodology is applicable to fabricate surface features over a range of sizes without any change in quality. Figure 6 shows the SEM images of densely packed, uniform, ordered arrangements of Si nanopillars over large areas of the substrate for all the materials sets used. Figure 6a reveals hexagonally ordered pillars with an average diameter of 38 nm and height of 150 nm for S1 after a 3 min Si etch time. The significant contrast enhancement in the inset of Figure 6a (compared to Figure 5a) suggests pattern transfer has occurred. The heights of the resultant patterns can be varied by changing the Si etch time without altering other processing conditions. At the longer etch times, well defined nanowire arrays can be formed. For S2, the nanopillars with heights 500 nm (Figure 6b) and 400 nm (inset of Figure 6b) are accomplished for 10 min and 8 min etch time respectively. Figures 6c, d and e tilted and cross sectional SEM images of the Si nanopillars/wires fabricated using S3, reveal Si nanowires with vertical smooth sidewalls of average heights about 250 nm, 400 nm and 500 nm for 5 min, 8 min and 10 min Si etch periods respectively. The average diameter of the nanopillars is around 18 nm in each case and remains almost equal throughout the length and little sign of narrowing or broadening effects are observed. Fig. 6f shows large area view of the pattern transferred substrate for S4, which demonstrate this protocol is applicable to a system with a dimension as small as 10 nm in diameter. Higher magnification tilted SEM image in the inset also reveal nanopillars with good sidewall profile with average diameter and height of 12 nm and 150 nm for 3 min Si etch time. No surface roughening or pattern damage is seen with decreasing the diameter or increasing the height of the pillars. Thus, ultra dense, high aspect ratio vertical silicon nanopillars/wires with a controlled placement and spacings over a large area can be realised by using iron oxide as a hard mask in the ICP etching process. Moreover, the mask can be easily removed by the oxalic acid aqueous solution without any pattern damage avoiding advanced and complicated processing steps.

### Magnetic studies of the iron oxide nanodot arrays

**Figure 7:**
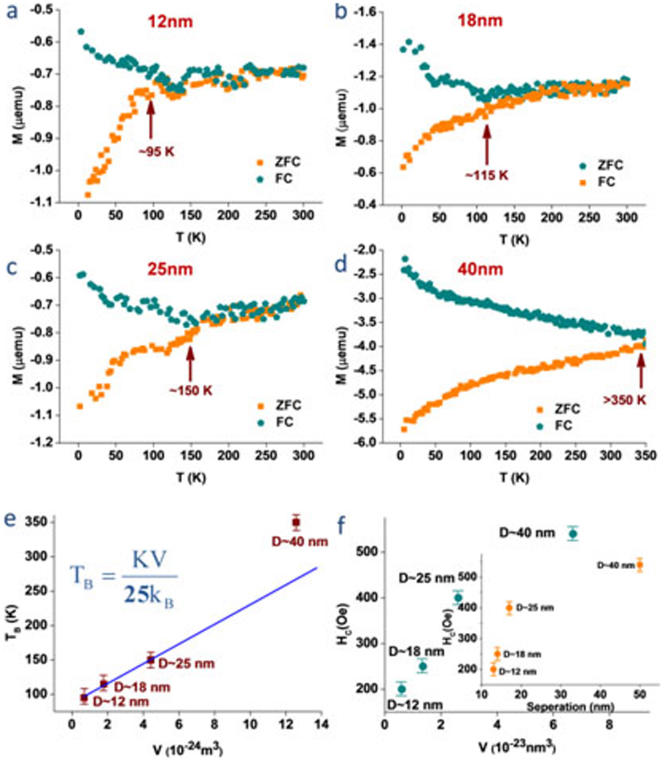
(a–d) Magnetization measurements as a function of temperature (MT) with an applied field of 100 Oe and temperature range 2 to 350 K using field cooling (FC) and zero-field cooling (ZFC) protocols for four different samples with different diameters from 12–40 nm. (e) The variation of T_B_ as a function of volume for individual nanodots is linear blue line is the fitted curve with equn. (T error bar 5 K). (f) The change of coercivity as a function of volume and separation (inset) for individual nanodots (H_C_ error bar 10 Oe).

These nanostructures prepared provide an ideal platform to quantify the magnetic properties of iron oxide nanodot assemblies of different sizes, particularly because not only they provide precise control in size and shape but also precise determination of separation. For quantitative estimation of magnetization, the samples were initially demagnetized with an appropriate protocol to remove any remnant magnetization. The orientation of the magnetization is an important parameter measured in-plane. The temperature dependences of field-cooled (FC) and zero-field cooled (ZFC) magnetization were measured with an applied magnetic field of 100 Oe from 350 K to 2 K for ordered array of Fe_2_O_3_ nanodots of different sizes (Figures 7a–d). The Magnetization-Temperature plots for different Fe_2_O_3_ nanodots array show a clear deviation of the zero-field cooled (ZFC) magnetization data from field-cooled (FC) magnetization on cooling which defines the blocking temperature (T_B_) for respective nanodots array of iron oxide. The average diameter (D) of the individual nanodots increases from 12 to 40 nm, where the thicknesses vary from 6–10 nm. The sizes of individual iron oxide nanodots (except 40 nm dot) are typical of sizes for single domain superparamagnetic iron oxide^12^,^27^. The magnetization vs. field (M-H) measurements was carried out to investigate the change of coercivity at 2 K for different diameter and separation (see supporting information). The blocking temperature (T_B_) increases linearly with the increase of diameter (D) of the nanodots until the dots reach a diameter of 25 nm (Figure 7e), following relation^28^,^29^,^30^ T_B_ = KV/25k_B_, where k_B_ is Boltzmann's constant, V is the volume of a single nanodot, and the effective anisotropy constant K. The linearity of the plot demonstrates the precise nature of the fabrication procedure and allows accurate determination of K. The nanodots diameters as well as the heights of the nanodots were considered to calculate the K value which increases with increasing the precursor concentrations and molecular weight of the block copolymer. The calculated value of K from the above equation for these iron oxide nanodots assemblies is 4.98 × 10^3^ J/m^3^ which is lower than the reported values for bulk magnetite 12 × 10^3^ J/m^3^ 29. The unusual decrease in magnetic anisotropy constant is due to the high surface to volume ratio and lack of crystallinity at the surface^31^. The coercivity of the ordered nanodots is measured for different dot sizes at 2 K and it decreases with the decrease of particle size (Figure 7f). The decrease in coercivity is due to dipolar interaction between well ordered magnetic particles^32^.

## Discussion

Previous works suggests that the polar PEO layer will preferentially wet the substrate surface (favourable PEO-substrate interactions) whilst PS will segregate to the air interface to form a PS-rich layer (PS has a lower surface energy, γ_PS_ = 33 mNm^−1^; γ_PEO_ = 43 mNm^−1^)^13^. The solvent/s chosen for solvent annealing depending on the molecular weight and weight fraction of PS (f_PS_) of the systems. The f_PS_ values for S1 and S3 are 0.75 and 0.744 are smaller compared to those for S2 and S4 which are 0.785 and 0.761 respectively. The equilibrium stability for vertical orientation were achieved by solvent annealing in toluene for the f_PS_ values less than or equal to 0.75 and toluene/water mixed solvent for higher values of f_PS_. Although PEO dissolves in toluene, it has larger solubility parameter difference with toluene, (δ_Tol_ – δ_PEO_ = 18.3−20.2 = 1.9 MPa^1/2^) than PS does (δ_Tol_ – δ_PS_ = 18.3−18 = 0.3 MPa^1/2^). Thus, toluene leads to a higher degree of swelling and imparts mobility to the PS chains. The vapour pressure of toluene at room temperature (0.0342 kPa) is not sufficient for the diffusion through the entire film thickness^33^ and an optimum temperature 50°C (toluene vapour pressure of 12.3 kPa) was used. Although 30 minutes of solvent exposure resulted in microphase separation but longer periods were needed to decrease the defect density to acceptable values. Care was exercised for the materials of higher PS weight fractions as the PEO cylinders underwent cyclical structural transitions between vertical and parallel orientation with annealing time^33^,^34^. In these cases, water was introduced as annealing co-solvent to achieve vertically oriented state of the final film because it reduces the surface segregation of PS.

A mechanism is proposed to create the nanoporous templates for the generation of oxide nanostructures. Ultrasonic treatment was a necessary criterion. We suggest that acoustic cavitation generates free radicals for bond cleavage of the PS-PEO. Generally, weak and strong acids are used for reconstruction or etching of these systems^35^,^36^. Here, ethanol was preferred as an etching solvent because of its' vapour pressure, selected solubility into PEO (since they have similar solubility parameters) and chemistry. Acoustic cavitation is more aggressive in solvents with a lower vapour pressure (5.95 kPa at 20°C)^37^. Further, the chemical structures of PEO monomers [(CH_2_CH_2_O)-] and ethanol molecules (H-CH_2_CH_2_O-H) are similar and it is asserted that during ultrasonication, PEO chains are surrounded by ethanol molecules allowing strong chemical interactions resulting in reaction and polymer degradation. After the bond cleavage, the PEO domains undergoes chemical interaction with ethanol at room temperature and becomes a miscible solution because at a very low PEO concentration, the solution possess a very low critical solution temperature (the volume fraction of PEO in ethanol is of the order of 2 × 10^−4^, assuming 1 ml. of PS-PEO solution was required for spin coating and the densities of PEO and toluene is 1.12 gm/cm^3^ and 0.865 gm/cm^3^ at room temperature)^38^. When ultrasound was ceased, the PEO molecules would prefer to separate from the solution but cannot because those PEO monomers and ethanol molecules cannot distinguish from each other, as a result, the PEO chains are frustrated and have no choice to form a crystalline layer when there is still some ethanol molecules present in the adjacent regions. Thus, the layer section consists of both ethanol and crystalline PEO as some ethanol is confined within the crystalline PEO layer^38^. This is why it is important to take out the film from the solution and dry it as quickly as possible. Two essential factors combined together facilitate the generation of nanoporous thin film with same transitional and orientational order. One is the orientation of the PEO cylinders normal to the film surface assists the diffusion of hydrophilic etching regent (Ethanol) to the PS/PEO interface. In addition, the glassy PS matrix can support the resultant nanoporous structure.

The nanoporous templates were used to create highly ordered iron oxide nanodots array by the selective inclusion of the metal cations through spin coating the precursor-ethanolic solution. The hydrophobic nature of PS prevents metal ion inclusion into the PS component whilst selective inclusion into the porous template is favoured by a combination of capillary forces and the affinity of PEO with the ionic solution. The spin coating procedure is highly efficient and it is suggested that the remaining PEO accelerates the metal ion inclusion process probably via either intra- or intermolecular coordination via electron donation from the PEO block to oxygen species in the ethanol molecule^13^. Considering the fact that the spin coating was performed for just a few seconds despite of different PS-PEO systems also support this principle. The wetting rate of one material on another is directly dependent on the dissolution kinetics which is related to the reactions occurring between them^39^. The gain in the free energy comes from the high reaction efficiency between PEO and metal cations are considered to create an additional driving force for the inclusion. Had complete removal of the PEO been achieved, it would be highly unlikely that significant metal uptake would occur because the PS matrix would be hydrophobic and the concentration of metal in solution is rather low.

This methodology of creation of iron oxide nanodots array allows dimensional and spatial control over a large substrate area. Also, high density, identical ordered, high aspect ratio Si nanopillars and nanowire arrays can be realized by using these iron oxide arrays as a hard mask material over silicon through ICP etch process. The methodology described here offers the advantage of high mask resolution on small feature sizes without mask-induced roughness or undesired sloping of the sidewalls. The relatively simple mask fabrication procedure with the standard existing etch recipes could significantly improve the manufacturing yield and reduce fabrication costs.

The magnetic studies suggest that the iron oxide nanoaprticles are superparamagnetic (except 40 nm), well isolated and size monodispersed. These precisely defined and size selective systems of ordered nanodots allow determining the linear relation of blocking temperature with diameter. We suggest that the inter-particle separation may also have a certain contribution on the magnetic counterpart. Presence of any inter-particle interaction alters the coherent rotation in magnetic reversal mechanism to an incoherent rotation and decreases coercivity^40^. The theoretical value of remanence over saturation magnetization (*M*_r_/*M*_s_) is 0.5 for non-interacting ferromagnetic particles^41^,^42^. As the inter particle dipolar interaction increases the remanence decreases. Thus this ratio decreases accordingly. The calculated value for *M*_r_/*M*_s_ in this case is 0.13–0.14 (12–40 nm) which is lower than 0.5 due to very low remanence. This confirms high level of dipolar interaction between these well order ferromagnetic nanodots. The inter particle distances (50, 17, 14 and 13 nm) decrease with size and enhance the dipolar interaction between neighbouring dots. At the distance below 20 nm due to stronger dipolar interaction the coercivity decreases rapidly and linearly with the separation (inset Figure 7f). This reduction both in remanence (*M*_r_) and coersivity (*H*_c_) occurs due to the demagnetizing field which arises from the generated magnetic dipoles formed at individual nanodots.

In conclusion, a simple solvent annealing approach was tuned to achieve long range hexagonal ordered vertical cylindrical microdomains for a wide range of diblock copolymer systems by controlling the solvents and the vapour pressure. An effective ethanol ultrasonication protocol for the degradation or modification of the PEO cylinders was developed to create templates for the generation of inorganic materials without pattern damage. Control over the size and spacing of iron oxide nanodots via a simple metal ion inclusion technique was demonstrated. The nanodots could be at sizes as low as 10 nm with an areal density of 6.7 × 10^11^ nanodots cm^−2^. Highly dense, large area, identical, hexagonally arranged Si nanopillar or nanowire arrays of smooth vertical sidewall profiles were fabricated by using these iron oxides as a hard mask. These silicon nanopillar and nanowire arrays demonstrate that this form of self-assembled, hardmask nanolithography can be an important component in the manufacturing of nanoscale devices with high throughput and low cost. The iron oxide nanodots demonstrated superparamagnetic properties with each dot being a single magnetic domain. The blocking temperature was linearly dependent with the size of the nanodots prepared here. The reliability and reproducibility of the techniques used here provide a method of generating sub 50 nm nanodots that might have a number of applications in fields as diverse as memory and biomedical applications.

## Methods

### Preparation of iron oxide nanodots by block copolymer inclusion technique

A series of asymmetric PS-b-PEO diblock copolymers, M_n_ = 102-34 kg mol^–1^, *M*_w_/*M*_n_ = 1.18; M_n_ = 42-11.5 kg mol^–1^, *M*_w_/*M*_n_ = 1.07; M_n_ = 32-11 kg mol^–1^, *M*_w_/*M*_n_ = 1.06; M_n_ = 16-5 kg mol^–1^, *M*_w_/*M*_n_ = 1.04 (where, *M*_n_ is the number-average molecular weight and *M*_w_ is the weight-average molecular weight) were purchased from Polymer Source. The PS-b-PEO systems are represented as S1, S2, S3 and S4 respectively. Single crystal B doped P type silicon (100) wafers (thickness 650 mm, resistivity 6–14 ohm cm) with a native oxide layer were used as a substrate. These were cleaned by ultrasonication in acetone and toluene for 30 min each and dried under nitrogen. 1 wt% polymer-toluene solutions were aged for 12 h at room temperature. PS-b-PEO thin films were fabricated by spin coating the polymer solution at 3000 rpm for 30 s onto Si wafers. The films were exposed to different solvents or a combination of solvents placed at the bottom of a closed vessel at a temperature 50°C to induce necessary chain mobility and allow microphase separation to occur. PS-PEOs (102-34) and (32-11) films were exposed to toluene for 2 h. Toluene/water (50:50, v/v) mixed vapour was used for the PS-PEOs (42-11.5) and (16-5) films under static vacuum for 1 h. Separate reservoirs were used for each solvent to avoid azeotropic effects. Partial etching and domain modification of PEO was carried out by ultrasonication of the films in anhydrous alcohol for different periods of time. After the desired time, the films were taken out from alcohol and dried immediately. For the fabrication of iron oxide nanodots, different concentrations of iron (III) nitrate nonahydrate (Fe(NO_3_)_3_,9H_2_O) in ethanol were spin-coated onto the nanoporous films. A UV/Ozone treatment was used to oxidize the precursor and remove the polymer. The as-prepared phase of iron oxide is Fe_3_O_4_, Fe_2_O_3_ nanodots were prepared by annealing them at 800°C for 1 h.

### Pattern transfer using ICP etch

These iron oxide nanodot arrays were used as a hard mask for pattern transfer to the substrate using an STS, Advanced Oxide Etch (AOE) ICP etcher as previously reported^21^. The system has two different RF generators, one, to generate and control the plasma density by direct connection to the antenna coil, while the other one was used to adjust and control the energy of the ions by connecting it to the substrate holder. A double etching process was used to, firstly, etch the native silica layer and, secondly, the silicon substrate. During etching, the sample is thermally bonded to a cooled chuck (10°C) with a pressure 9.5 Torr. For the oxide layer etch, the process parameters were optimised to a C_4_F_8_/H_2_ gas mixture (21 sccm/30 sccm) using an ICP coil power of 800 W and a Reactive Ion Etching (RIE) power of 80 W. The silica etch time was kept constant (10 sec) for all the samples. For Si pillar fabrication, the process used a controlled gas mixture of C_4_F_8_/SF_6_ at flow rates of 90 sccm/30 sccm respectively and the ICP and RIE power were set to 600 W and 15 W respectively at a chamber pressure of 15 mTorr.

### Characterizations

Surface morphologies were imaged by scanning probe microscopy (SPM, Park systems, XE-100) in tapping mode and scanning electron microscopy (SEM, FEI Company, FEG Quanta 6700). The film thicknesses were measured by optical ellipsometer (Woolam M2000) and electron microscopy. The ordering of the film was investigated by grazing incidence small angle X-ray scattering (GISAXS). The measurements were performed with a diamond light source at beamline I07 (SI 8065) with monochromatized X-rays (λ = 0.15 nm) having grazing incident angle ranging from 0.09 to 0.20°. Samples were prepared for TEM cross sectional imaging with an FEI Helios Nanolab 600i system containing a high resolution Elstar™ Schottky field-emission SEM and a Sidewinder FIB column and were further imaged by transmission electron microscopy (TEM, JEOL 2100). X-Ray photoelectron spectroscopy (XPS) experiments were conducted on a Thermo K-alpha machine with Al K_α_ X-ray source operating at 72 W. FTIR spectra were recorded on infrared spectrometer (IR 660, Varian). The magnetic properties of the samples were investigated using a Superconducting Quantum Interference Device (Model: Quantum Design MPMS-XL5).

## Author Contributions

T.G. developed the synthesis methods, performed experiments and characterizations, analysed data, wrote the article. T.M. and S.R. performed magnetic measurements, analysed and wrote the corresponding part. R.S. performed the scanning electron microscopy. M.T.S. developed and performed pattern transfer protocol. P.C. performed the FIB thinning and transmission electron microscopy. M.A.M. And J.D.H. conceives the idea and supervised the study and the article. All authors discussed the results and commented on the article.

## Supplementary Material

Supplementary Information Supporting Information
