# Supplementary material for: Size and space controlled hexagonal arrays of superparamagnetic iron oxide nanodots: magnetic studies and application
Source: Sci Rep. 2013 Sep 27;3:2772. doi: 10.1038/srep02772 (PMC3784943; doi:10.1038/srep02772)
Supplement: Supplementary Information — Supporting Information [file srep02772-s1.doc]

Size and space controlled hexagonal arrays of superparamagnetic iron oxide nanodots: magnetic studies and application

*Tandra Ghoshal,^1,2^ Tuhin Maity,^3^ Ramsankar Senthamaraikannan,^1,2^ Matthew. T. Shaw,^4^ Patrick Carolan,^1^ Justin D. Holmes,^1,2^ Saibal Roy,^3, †^ Michael A. Morris^1,2^**

^1^Materials Research Group, Department of Chemistry and Tyndall National Institute, University College Cork, Cork, Ireland

^2^Centre for Research on Adaptive Nanostructures and Nanodevices (CRANN), Trinity College Dublin, Dublin, Ireland

^3^Micropower-Nanomagnetics group, Tyndall National Institute, Cork, Ireland

^4^Intel Ireland Ltd., Collinstown Industrial Estate, Co. Kildare, Ireland

*^†^*Present Address: Electrical Engineering Department, Stanford University, California, USA (on sabbatical from Tyndall National Institute, Ireland)

**Supporting Information:**

**Determination of phases of iron oxide nanodots before and after annealing.**

XPS was used to confirm the crystalline phase of the iron oxide nanodots on Si substrate after UV/Ozone treatment and further calcination. High resolution Fe2p spectra were recorded to distinguish different phases of iron oxides. Fe 2p core level spectrum recorded on iron oxide nanodots prepared after UV/Ozone treatment (Figure 1a) consists of two peaks associated with Fe 2p_3/2_ at 711 eV and Fe 2p_1/2_ at 724.4 eV and broadened due to the existence of Fe^+2^ and Fe^+3^ ions. The Fe 2p_3/2_ and Fe 2p_1/2_ binding energies (BEs) for Fe^+2^ and Fe^+3^ were determined by curve-fitting using Gaussian-Lorentzian line shapes. The measured Fe 2p_3/2_ and Fe 2p_1/2_ BEs are 709.7 and 723 eV (assigned to Fe^+2^) and 711.6 and 725 eV (Fe^+3^) matches literature values.^1^ The concentration ratio of Fe^+3^/ Fe^+2^ was calculated from the curve-fitted peak areas as about 2:1 as expected for Fe_3_O_4_. Fe 2p core level spectrum of iron oxide nanodots after calcination (Figure 1b) consists of two sharp peaks associated with Fe 2p_3/2_ and Fe 2p_1/2_ at 711.3 and 725.1 eV accompanied by high binding energy satellite structures (+8 eV shift). These data are consistent with the existence of Fe^+3^ (Fe_2_O_3_) ions only.^2-3^

**Figure 1** High resolution spectrum for Fe 2p core level revealed (a) Fe_3_O_4_ and (b) Fe_2_O_3_ phase.

**Determination of crystalline structure of iron oxide nanodots before and after annealing by TEM.**

The crystalline information of these systems is exemplified further by TEM (Figure 2). The nanodots on Si substrate were scratched by a sharp edge blade and disperse into ethanol for the preparation of TEM grid. Clear lattice fringes can be seen from Figure 2a revealed the single crystalline nature of the UV/Ozone treated sample. The lattice fringes were regularly separated with a spacing of 0.2967 nm, which agrees well with the (220) lattice index of cubic Fe_3_O_4_ (Figure 2a).[^4^](#_ENREF_29) Similarly, the single crystalline nature of the high temperature calcined sample was revealed by the HRTEM image (Figure 2b). Lattice fringes were regularly separated at 0.252 nm agreeing with the (110) lattice spacing of rhombohedral hematite.^5^


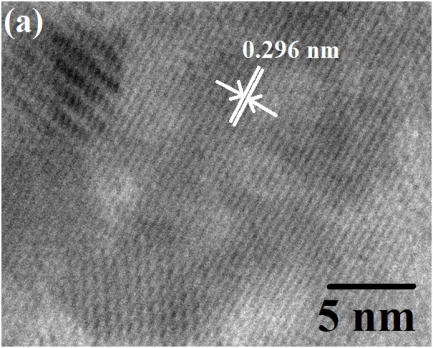

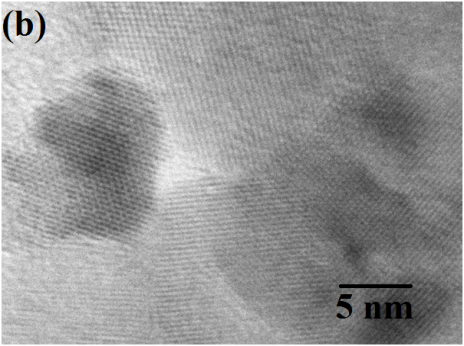

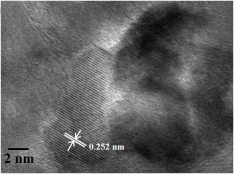


**Figure 2** HRTEM image of (a) Fe_3_O_4_ and (b) Fe_2_O_3_ nanodots. Inset of (b) shows corresponding fringe spacings from nanodots.

**M-H measurements.**

The magnetization Vs. field (M-H) measurements were carried out on Fe_2_O_3_ nanodots. The diamagnetic contribution from the quartz substrates was estimated, subtracted from the directly measured results shown in Figure 3.

**Figure 3** M-H curve of different diameter Fe_2_O_3_ nanodots.

1. Prakash, R., Choudhary, R. J., Chandra, L. S. S., Lakshmi, N. & Phase, D. M. Electrical and magnetic transport properties of Fe3O4 thin films on a GaAs(100) substrate. *J. Phys.-Condes. Matter* **19** (2007).

2. Mills, P. & Sullivan, J. L. A study of the core level electrons in iron and its 3 oxides by means of x-ray photoelectron-spectroscopy. *J. Phys. D-Appl. Phys.* **16**, 723-732 (1983).

3. Fujii, T. *et al.* In situ XPS analysis of various iron oxide films grown by NO_2_-assisted molecular-beam epitaxy. *Physical Review B* **59**, 3195-3202 (1999).

4. Hui, C. *et al.* Large-scale Fe_3_O_4_ nanoparticles soluble in water synthesized by a facile method. *J. Phys. Chem. C* **112**, 11336-11339 (2008).

5. Chen, Z. Q., Cvelbar, U., Mozetic, M., He, J. Q. & Sunkara, M. K. Long-range ordering of oxygen-vacancy planes in alpha-Fe_2_O_3_ nanowires and nanobelts. *Chem. Mat.* **20**, 3224-3228 (2008).
